# "*Summary Page*": a novel tool that reduces omitted data in research databases

**DOI:** 10.1186/1471-2288-10-91

**Published:** 2010-10-08

**Authors:** Saveli I Goldberg, Andrzej Niemierko, Maria Shubina, Alexander Turchin

**Affiliations:** 1 Massachusetts General Hospital, Boston, MA, USA; 2 Brigham and Women's Hospital, Boston, MA, USA; 3 Clinical Informatics Research and Development, Partners HealthCare, Boston, MA, USA; 4 Harvard Medical School, Boston, MA, USA

## Abstract

**Background:**

Data entry errors are common in clinical research databases. Omitted data are of particular concern because they are more common than erroneously inserted data and therefore could potentially affect research findings. However, few affordable strategies for their prevention are available.

**Methods:**

We have conducted a prospective observational study of the effect of a novel tool called "*Summary Page*" on the frequency of correction of omitted data errors in a radiation oncology research database between July 2008 and March 2009. "*Summary Page*" was implemented as an optionally accessed screen in the database that visually integrates key fields in the record. We assessed the frequency of omitted data on the example of the *Date of Relapse *field. We considered the data in this field to be omitted for all records that had empty *Date of Relapse *field and evidence of relapse elsewhere in the record.

**Results:**

A total of 1,156 records were updated and 200 new records were entered in the database over the study period. "*Summary Page*" was accessed for 44% of all updated records and for 69% of newly entered records. Frequency of correction of the omitted date of cancer relapse was six-fold higher in records for which "*Summary Page*" was accessed (p = 0.0003).

**Conclusions:**

"*Summary Page*" was strongly associated with an increased frequency of correction of omitted data errors. Further, controlled, studies are needed to confirm this finding and elucidate its mechanism of action.

## Background

Data entry errors are common in clinical research studies[1-3]. Omitted data are particularly significant because they are more prevalent than erroneously inserted data and are therefore more likely to lead to errors in analysis and conclusions[4]. Statistical approaches have been developed to analyse datasets with missing data, such as multiple imputation and mixed-effects regression models[5-8]. However, these methods are applicable only to data with "missing at random" (MAR) pattern of missingness, while errors of omission (interpreted as missing data) usually do not satisfy the MAR requirements. Consequently, omitted data can have a significant effect on research findings[9].

Reduction in frequency of data errors is important in order to ensure high quality of data used in clinical research and the resulting conclusions. However, double-entry - the gold standard approach for prevention of data errors - is labor-intensive and costly[10]. Other proposed approaches such as direct patient contact and more rigorous documentation and/or communication policies may also be labor-intensive and/or not explicitly validated[11]. It has been shown that the probability of errors increases in cognitively isolated database fields in the database record - fields, whose content is independent of the other fields in the record[12]. For example, the date of the first radiation treatment commonly takes place soon after the date of diagnosis, but the date of the last follow-up visit can take place any time after the diagnosis and is therefore more error-prone. Based on this finding, we have developed a novel tool called "*Summary Page*" that decreases the incidence of errors in clinical research databases by enhancing cognitive connectivity between the fields in the database record. In this study we evaluated the effect of the "*Summary Page*" access on the frequency of correction of omitted data in research databases.

## Methods

### Design

We conducted prospective observational analysis of data entered in a radiation oncology clinical research database to determine whether utilization of the "*Summary Page*" was associated with higher frequency of correction of omitted date of cancer relapse (primary outcome).

### Data Sources

We analysed the data from two research databases at the Department of Radiation Oncology at an academic medical centre: a) breast cancer treatment database (database B) and b) sarcoma treatment database (database S). Both databases contained information about cancer treatment (including surgery, chemotherapy and radiation) of oncologic patients who underwent radiation treatment at the department. Data in these databases were entered retrospectively and the databases were completely separate from the electronic medical record systems utilized for clinical care. All data in the databases were entered manually by trained technicians, usually being copied from electronic or paper medical records. A typical record contained the patient's demographics and, information about the diagnosis, co-morbidities, treatment and outcomes. Database B has 11 data entry forms and database S has 14 data entry forms for each record.

### Analysis of Omitted Data

We analysed the data in database S to determine prevalence and co-occurrence of omitted data. In order to distinguish between the data that was truly missing and omission errors we identified all records that did not have any information in the field XRT (X-ray Therapy) Start Date. These records were subsequently manually reviewed to obtain XRT Start Date where it could be found. We then identified records that had missing information in fields where it would most likely be due to an error of omission including the patient's gender, date of birth (DOB) and cancer site. We compared the prevalence of missing data in these three fields in the following categories of records: a) records with no missing XRT Start Date; b) records with missing XRT Start Date for which this information could not be found on manual review (i.e. was not the result of an omission error); and c) records with missing XRT Start Date for which this information was subsequently found on manual review (i.e. was the result of an omission error).

### "*Summary Page*" Tool

**Figure 1:**
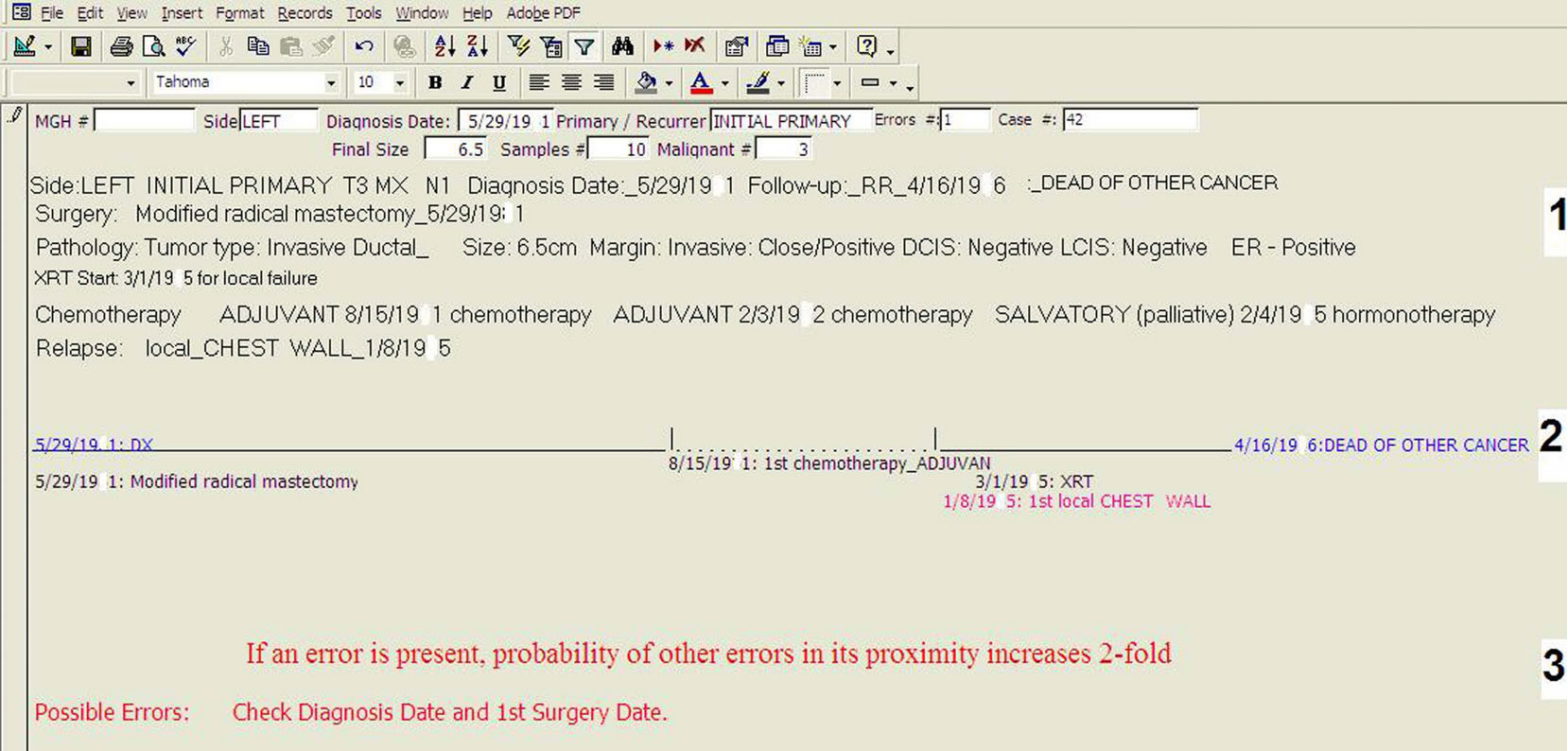
***Summary Page*" screen in the database**. "*Summary Page*" is implemented as a single screen in Microsoft Access database that has the following major components. 1. Verbatim listing of selected fields in the patient's record representing key demographic and clinical information. 2. Schematic representation of the timeline of the patient's clinical course. 3. "Possible Error" section that lists likely errors based on integrated analysis of all fields in the records. Typically these errors while likely, are not fully certain, and therefore do not merit an interruptive alert

**Figure 2:**
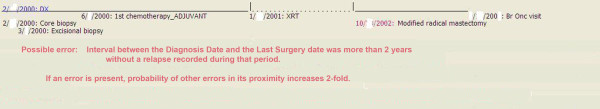
**Example of a possible error highlighted by the "*Summary Page*"**. This breast cancer patient was treated with lumpectomy (excisional biopsy) followed by XRT. Subsequently after no events are recorded for year and a half she has a modified radical mastectomy. This could indicate a possible error (e.g. omitted date of cancer relapse). However, the error is not definite as sometimes patients request a more comprehensive surgery on their own without overt clinical indications.

The "*Summary Page*" tool was developed to exploit the fact that errors are more likely to occur in data fields that are not cognitively connected to any of the other fields in the database. It is implemented as a single screen that has the following major sections (Figure 1):

1. Verbatim listing of selected fields in the record representing key demographic and clinical information.

2. Schematic representation of the timeline of the patient's clinical course

3. "Possible Error" section that lists likely errors based on a set of rules that take into account all fields in the record. Typically these errors, while likely, are not fully certain, and therefore do not merit an interruptive alert. An example of a possible error that can be identified in the breast cancer database is a new surgical procedure (e.g. modified radical mastectomy after the original lumpectomy) without a documented relapse of the cancer (Figure 2). This is most likely an error where the information about the relapse was omitted. However, the error is not definite since it is also possible that the patient herself requested a more aggressive procedure in order to be reassured that the cancer will not recur. Most rules for identification of possible errors involve data elements entered on different screens of the database which may therefore be more difficult for the users to integrate. In the described implementation of the "Summary Page" these rules were based on expert opinion but they can potentially also be derived using formal algorithms, including supervised and non-supervised learning techniques. A full set of "possible error" rules utilized in the described implementation of the "*Summary Page*" is listed in the Additional File 1.

"*Summary Page*" aims to reduce the incidence of data errors through the following mechanisms:

a) Enhancement of cognitive links between the key fields in the database record through visual integration on a single screen

b) Identification of possible, though not fully certain, errors through integrated analysis of the data. For example, unexpected treatment activity can be a sign of missing relapse information

The "*Summary Page*" was implemented as a single page in Microsoft Access database that could be optionally accessed from anywhere in the record. Users can access "*Summary Page*" any time during data entry process or never access it at all.

### "*Summary Page*" Utilization and Effectiveness

In order to evaluate the effectiveness of the "*Summary Page*" tool in reducing the frequency of omitted data we analyzed the changes to the database records and "*Summary Page*" user tracings in database "B" between 07/04/2008 and 03/04/2009. We assessed the frequency of omitted data on the example of the *Date of Relapse *field. We considered the data in this field to be omitted for all records that had empty *Date of Relapse *field and evidence of relapse elsewhere in the record.

"*Summary Page*" usage tracings included date, time and user ID for each "*Summary Page*" access. We also recorded date, time, user ID, and before and after values for each edit of the *Date of Relapse *field. Database users were not aware that "*Summary Page*" access was being monitored.

### Statistical Analysis

Summary statistics were constructed by using frequencies and proportions for categorical data. Reported confidence limits (CI) for proportions are exact binomial CI. Fisher's Exact Test was used for analyses of bivariate associations between categorical variables. To identify the predictors of "*Summary Page*" access and entry of an omitted date of relapse we constructed hierarchical (multilevel) multiple logistic regression models. Both models adjusted for clustering within individual database users using repeated effects method. Significance thresholds were adjusted for multiple hypothesis testing using Simes-Hochberg method[13,14]. All analyses were performed using SAS (Version 9.1; SAS, Cary, NC). All statistical tests were 2-sided.

### IRB

The study protocol was reviewed and approved by the Partners Human Research Committee.

## Results

### Data entry omission errors

Database S contained 2,250 records of patients treated with radiation therapy (XRT) for sarcoma. Of these, 344 records did not contain information about XRT start date and were manually re-checked to determine whether documentation of the XRT start date could be found in the patient's records. As a result of manual verification, XRT start date was identified for 118 (34.3%) records.

Missing data in other fields (Gender, DOB, Cancer Site) was most common among records with missing XRT start date that was found on manual review (at least one of the three fields was missing in 34.7% of the records) and least common among records (7.6%) where XRT start date was not missing or the missing XRT start date could not be subsequently identified on manual verification (p < 0.0001). The difference between the prevalence of missing data in other fields between records with correctly vs. erroneously missing XRT start date was also highly significant (Table 1).

**Table 1:** Prevalence and Co-Occurrence of Missing Data

	Total	Missing Gender, N (%, 95% CI)	Missing DOB, N (%, 95% CI)	Missing Cancer Site N (%, 95% CI)
Record with missing XRT Start not found on manual review (no data error)	226	18 (8.0; 4.8-12.3)	15 (6.6; 3.8-10.7)	20 (8.85; 5.5-13.3)

Records with missing XRT Start found on manual review (data error: omitted XRT start)	118	18 (15.2; 9.3-23.0)	12 (10.2; 5.4-17.1)	24 (20.3; 13.5-28.7)

P-value^1^		< 0.0001	0.031	< 0.0001

^1^P-value represents a Fisher exact test between the fractions of records with omitted data among records with omitted vs. not omitted XRT start date

### "*Summary Page*" Utilization

**Figure 3:**
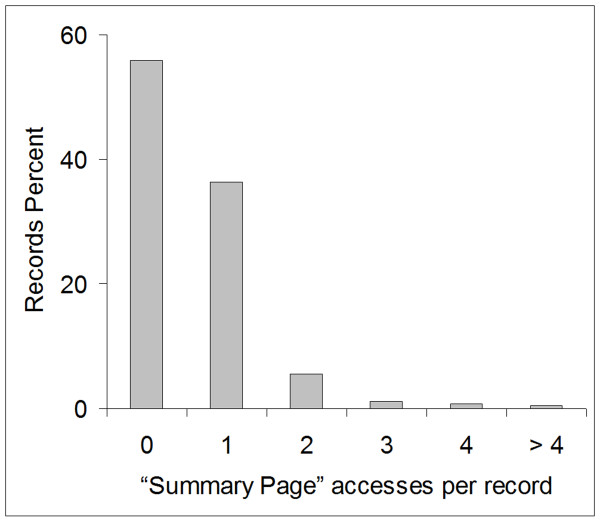
**"*Summary Page*" Utilization per Record**.

To determine the utilization of the "*Summary Page*", we analysed all 1356 records (200 new and 1156 updated) that were entered or updated in database B between 07/04/2008 and 03/04/2009. Distribution of "*Summary Page*" utilization per record is presented in Figure 3. "*Summary Page*" was accessed in slightly less than half (44.2%) of all records. Most commonly it was accessed only once per record but in 2.4% of the records it was accessed three or more times. "*Summary Page*" was accessed for 69.0% of entries of new records but only for 40.0% of the updates of the records already in the database (p = 0.01). Three data entry technicians worked with the database during this period. Their "*Summary Page*" access rate was 84.6%, 47.1% and 11.8%, respectively (p < 0.001).

In multivariable analysis the odds of "*Summary Page*" access were 12% higher if the patient's cancer had T stage greater than 1 and over 50% higher if this was a new record rather than an existing one being updated (Table 2). Patients' vital status, tumor grade, whether the cancer had already been recorded as having relapsed prior to the record update being analyzed, and the year of diagnosis were not significantly associated with "*Summary Page*" access.

**Table 2:** Predictors of "*Summary Page*" Access

Predictor Variable	Odds of "*Summary Pag*e" Access	P-value
Deceased	0.99	0.73

Cancer Relapsed	0.94	0.23

Tumor grade "poor"	1.03	0.37

T stage > T1	1.12	0.0018

Diagnosis Year	1.0	0.78

New case	1.51	< 0.0001

### "*Summary page*" Effectiveness

To evaluate the effectiveness of the "*Summary Page*" we analysed 1356 records (200 new and 1156 updated) that were entered or updated between 07/04/2008 and 03/04/2009. Among these, 164 had documented a remission but had final vital status "Died from Disease", indicating that a relapse had taken place. Five of these 164 records (3.05%) did not have any information in the *Date of Relapse *field recorded. "*Summary Page*" was accessed for 86 of the 164 records. None of the records where "*Summary Page*" was accessed had a missing date of relapse (p = 0.023).

Among the 1,156 records that were updated during the study period, "*Summary Page*" was accessed for 462 records. Relapse information was entered after "*Summary Page*" access for 16 (3.5%) of the records where "*Summary Page*" was utilized and for 4 (0.6%) of the remaining records (p = 0.0003). In all 16 records where relapse information was entered after "*Summary Page*" access, it was entered within 10 minutes of accessing the "*Summary Page*" (typically a single record takes 20-90 minutes to enter de novo and 10-40 minutes to update). Similarly, out of the 9 records where the date of the very first relapse was entered, it was entered within 10 minutes after "*Summary Page*" access for 7 records (p = 0.034). In multivariable analysis the odds of entry of a date of relapse increased by 3.8% if the patient's vital status was "Deceased" and by 4.5% if the "*Summary Page*" was accessed during the record update (Table 3). Tumor grade, tumor stage and the year of cancer diagnosis were not significantly associated with the probability of entry of a date of relapse. There was no significant difference of probability of entry of a relapse date between individual data entry technicians.

**Table 3:** Predictors of Relapse Date Entry

Predictor Variable	Odds of Relapse Date Entry	P value
Deceased	1.038	0.0026

Tumor grade "poor"	1.006	0.58

T stage > T1	1.008	0.48

Diagnosis Year	1.0	0.79

"*Summary Page*" accessed	1.045	< 0.0001

## Discussion

In this prospective observational study we showed that utilization of a novel tool - "*Summary Page*" - is associated with lower rates of omitted data in research databases.

Many types of errors are common in research databases[1,2,12,15]. However, omitted data constitute a particularly perilous type of error because they are much more common than erroneously inserted information. Non-MAR/non-MCAR type errors of omission in turn are more likely to affect the conclusions of analysis. This is well illustrated by the error on which we focused our evaluation - omitted date of cancer relapse. This information is not commonly found as a structured field in electronic medical record systems. Therefore it has to be manually abstracted from the patient's record by trained personnel through interpretation and cognitive integration of multiple types of narrative documents, including progress notes, operative reports, imaging studies, pathology reports, etc. Consequently, information about a relapse date would be easy to miss. On the other hand, erroneous entry of a non-existing relapse date is unlikely. Hence, introduction of wrong data and omission of correct data have different probabilities. Whereas omitted data in fields containing continuous variables that are MAR with respect to the information available in the record might be successfully dealt with by imputing values based on the other fields in the record, omitted date of relapse would most likely be interpreted as a lack of relapse, potentially altering study conclusions.

Many contemporary electronic data entry systems (e.g. online stores or banks) allow the user to review the information they had entered prior to finalizing the transaction. However, complete data review is not feasible for clinical research databases whose records typically have dozens if not hundreds of fields that take up multiple screens. The "*Summary Page*" is designed to mitigate this problem by applying the following principles: a) focusing on the key fields; b) visually integrating information in these fields to facilitate detection of discrepancies and contradictions; and c) pointing out probably (though not necessarily definite) errors.

In order to help the user identify possible errors "*Summary Page*" aims to facilitate cognitive connections between different fields in the database by showing them together on a single screen. For example, "*Summary Page*" displays both the date of relapse and the patient's treatment dates. If, for example, the patient had chemotherapy several years after mastectomy but there is no date of relapse recorded between the surgery and the chemotherapy, the omission becomes very apparent to the user. Furthermore, as we showed in the analysis of prevalence of omitted data, errors of omission frequently co-occur in the same record. A user who has identified one error of omission in the record may also be more likely to review the rest of the record for other omitted data. Therefore the increased frequency of correction of omitted data we observed in the "*Summary Page*" users can be due to several mechanisms.

A number of approaches have been used to reduce errors in research databases. Double-entry of the same information by multiple personnel followed by verification of all discrepancies remains the gold standard but is expensive. Constraint failure alerts are less costly. However, not all fields have obvious and persistent constraints, limiting applicability of this approach. "*Summary Page*" is a method that is at the same time more flexible than constraint failure and is not labour intensive. Importantly, "*Summary Page*" was well accepted by the database users. Despite being completely optional, it was used in nearly half of all records and in over two thirds of the new records. "*Summary Page*" could therefore serve as a complement to double entry to improve its efficiency (by reducing the number of errors to be reconciled between two entries) or as a means to reduce the number of errors under circumstances where double entry is not feasible. Making accessing "*Summary Page*" mandatory (e.g. at the end of the editing session for a particular record) could further increase its impact on error rates.

"*Summary Page*" may not be a perfect tool for all settings. It is less likely to be useful for smaller databases where entering a single record requires only 1-2 screens. Under these circumstances its benefit of integrating information from distant parts of the record may be lower. "*Summary Page*" is also not a tool that can be used off-the-shelf. It must be set up individually for each database by qualified personnel who may not be available or may require additional expense on consultants. Optimal set-up of the "*Summary Page*" requires knowledge of the subject matter and relevant workflow. For example, unexpected therapeutic activity (e.g. surgery) may indicate a missing cancer relapse information whereas abrupt discontinuation of radiation therapy may be a sign of omitted information about radiation toxicity.

Utilization of the "*Summary Page*" differed significantly between the three users, although the low overall number of the users precludes drawing statistical conclusions. The individual with the lowest (11.8%) rate of utilization of the "*Summary Page*" was a visiting physician. Data entry was not one of that individual's direct duties and it is possible that this decreased overall motivation. The individual with the highest (84.6%) rate of utilization was only involved in data entry over a short period of time immediately after the "*Summary Page*" was introduced. It is possible that the novelty of the technology had contributed to the higher rate of utilization. The third individual was the only one who was involved in the data entry into the study database throughout the study period and was an experienced data entry technician in the department. This individual's rate of utilization (47.1%) is representative of the utilization rates we had observed in day-to-day use of the "*Summary Page*" in the department.

Optimal database design remains an important tool for error prevention. The *Date of Relapse *field we analyzed in this study had overloaded the *NULL *value, which could mean either that there was no date of relapse (i.e. the cancer had not relapsed) or that the date of relapse was not known (e.g. there was information that the cancer had relapsed, but the exact date when the relapse was diagnosed could not be established). Availability of a distinct *Unknown *value for the *Date of Relapse *field would have likely also decreased the rate of omitted date of relapse observed in our study.

Our study had several limitations. Its observational nature and lack of a control group does not allow us to draw direct inference about the effectiveness of the "*Summary Page*". While the difference between the number of omitted relapse date values entered with vs. without the use of the "*Summary Page*" was large and highly significant, we do not have information about all omitted relapse dates in the dataset and therefore cannot rule out that these were distributed unequally between the records where "*Summary Page*" was vs. was not used, accounting for all or part of this difference. The effect of the "*Summary Page*" was evaluated on the example of a single field in one radiation oncology research database. It is therefore possible that it may not be as effective in other settings. "*Summary Page*" may require user training to achieve its full potential. For example, it is unlikely to be helpful if accessed in the beginning of the data entry session when there is little data in the record, but much more helpful in the end.

## Conclusions

In this prospective observational study we have found that data omission errors in clinical research databases were common and non-randomly distributed. Utilization of a novel tool, "*Summary Page*", was strongly associated with an increase in the frequency of correction of omitted data. Further (e.g. randomised controlled) studies are needed to confirm this finding and establish the mechanism of action of the "*Summary Page*".

## Competing interests

The authors of this manuscript have no conflicts of interest. There are no plans to market or sell the system described in the manuscript.

## Authors' contributions

SIG designed and implemented the Summary Page tool, carried out the evaluation and drafted the manuscript. AN participated in the study design and critical revision of the manuscript. MS assisted with bio-statistical analysis, study design and critical revision of the manuscript. AT participated in the study design, analysis and interpretation of data and critical revision of the manuscript.

## Pre-publication history

The pre-publication history for this paper can be accessed here:

http://www.biomedcentral.com/1471-2288/10/91/prepub

## Supplementary Material

Additional file 1 **Probable Error Rules**. A complete listing of "possible error" rules utilized in the described implementation of the "*Summary Page*". Click here for file

## References

[B1] Beretta L Aldrovandi V Grandi E Citerio G Stocchetti N Improving the quality of data entry in a low-budget head injury database Acta Neurochir (Wien) 2007 149 9 903 909 10.1007/s00701-007-1257-3 17665088

[B2] Seddon DJ Williams EM Data quality in population-based cancer registration: an assessment of the Merseyside and Cheshire Cancer Registry Br J Cancer 1997 76 5 667 674 9303369 10.1038/bjc.1997.443 PMC2228008

[B3] Arts DG De Keizer NF Scheffer GJ Defining and improving data quality in medical registries: a literature review, case study, and generic framework J Am Med Inform Assoc 2002 9 6 600 611 10.1197/jamia.M1087 12386111 PMC349377

[B4] Molenberghs G Kenward M Missing Data in Clinical Studies 2007 Hoboken, NJ: Wiley

[B5] D'Agostino RB Jr Rubin DB Estimating and using propensity scores with partially missing data Journal of the American Statistical Association 2000 749 759 10.2307/2669455

[B6] Hedeker D Gibbons RD Application of random-effects pattern-mixture models for missing data in longitudinal studies Psychological Methods 1997 2 1 64 78 10.1037/1082-989X.2.1.64

[B7] Friedman LM Furberg C DeMets DL Fundamentals of clinical trials 1998 3 New York: Springer

[B8] Good PI A manager's guide to the design and conduct of clinical trials 2006 2 Hoboken, N.J.: Wiley-Liss

[B9] Goldhill DR Sumner A APACHE II, data accuracy and outcome prediction Anaesthesia 1998 53 10 937 943 10.1046/j.1365-2044.1998.00534.x 9893535

[B10] Day S Fayers P Harvey D Double data entry: what value, what price? Control Clin Trials 1998 19 1 15 24 10.1016/S0197-2456(97)00096-2 9492966

[B11] Wisniewski SR Leon AC Otto MW Trivedi MH Prevention of missing data in clinical research studies Biological Psychiatry 2006 59 11 997 1000 10.1016/j.biopsych.2006.01.017 16566901

[B12] Goldberg S Niemierko A Turchin A Analysis of data errors in clinical research databases AMIA Annu Symp Proc 2008 242 246 18998889 PMC2656002

[B13] Simes RJ An improved Bonferroni procedure for multiple tests of significance Biometrika 1986 73 3 751 754 10.1093/biomet/73.3.751

[B14] Hochberg Y A sharper Bonferroni procedure for multiple tests of significance Biometrika Trust 1988 75 800 802 10.1093/biomet/75.4.800

[B15] Shelby-James TM Abernethy AP McAlindon A Currow DC Handheld computers for data entry: high tech has its problems too Trials 2007 8 5 10.1186/1745-6215-8-5 17309807 PMC1804282

