# Supplementary material for: "Summary Page": a novel tool that reduces omitted data in research databases
Source: BMC Med Res Methodol. 2010 Oct 8;10:91. doi: 10.1186/1471-2288-10-91 (PMC2964731; doi:10.1186/1471-2288-10-91)
Supplement: Additional file 1 — Probable Error Rules. A complete listing of "possible error" rules utilized in the described implementation of the "Summary Page". [file 1471-2288-10-91-S1.DOC]

Appendix 1

Probable Error Rules

| **Rule** | **Explanation** |
| --- | --- |
| XRT end date more than 1.5 months after the XRT start date | Radiation treatment does not usually last more than 1.5 months (typically < 1 month) |
| Number of business days between XRT start and XRT end dates less than the number of fractions (treatments) | Radiation treatments are usually only administered on business days (not on weekends or public holidays). |
| Total Dose / # of fractions > 2.1 Gy | A single fraction (treatment) usually provides less than 2.1 Gray of radiation |
| Mastectomy Date less than 2 weeks after the Diagnosis Date | Mastectomy is usually done more than 2 weeks after the initial diagnosis date (to allow for time necessary for biopsy processing etc.) |
| XRT Start Date less than 3 weeks after the Diagnosis Date | Radiation treatment is usually started more than 3 weeks after the initial diagnosis (to allow for time necessary for biopsy processing and surgical excision). |
| Chemotherapy Start Date less than 3 weeks after the Diagnosis Date | Chemotherapy treatment is usually started more than 3 weeks after the initial diagnosis (to allow for time necessary for biopsy processing and surgical excision). |
| T-stage = 2 and Tumor Size < 2 cm | Breast cancer staging guidelines suggest that T2 be assigned primarily to tumors ≥ 2 cm in size |
| T-stage = 3 and Tumor Size < 5 cm | Breast cancer staging guidelines suggest that T3 be assigned primarily to tumors ≥ 5 cm in size |
| Adjuvant Chemotherapy End date more than 2 years after the Start date | Adjuvant chemotherapy for breast cancer (initial chemotherapy following surgical excision) usually lasts less than 2 years |
| Cycles of adjuvant chemotherapy > 6 | Adjuvant chemotherapy is usually delivered in 6 or fewer cycles |
| XRT Start more than 6 months after last surgery | Radiation treatment is usually started soon after surgical excision of the tumor |
| Chemotherapy start more than 6 months after last surgery without relapse | Chemotherapy is usually started soon after surgical excision of the tumor |
| Chemotherapy start more than 6 months after XRT End Date without relapse | Chemotherapy is usually started soon after completion of radiation therapy |
| Last surgery more than 2 years after the initial diagnosis date without any recorded relapse during this period of time | It would be unusual to have a surgery if the cancer had not relapsed. |
| Margin = Negative and Total Dose > 64 Gy | It is unusual for total radiation dose to exceed 64 Gy if the tumor excision was complete (negative margins) |
| Tumor Size > 5 cm, Age > 50 and Lumpectomy | Lumpectomy is usually performed in women younger than 50 who have a tumor less than 5 cm in size |
| Age < 30 | It is uncommon to have breast cancer before the age of 30 |
| Male | It is uncommon for a man to develop breast cancer. |
| Tangents (radiation directed immediately at the tumor) Dose < 40 or > 51 Gy | It is uncommon for the tangents radiation dose to be < 40 or > 50 Gy |
| Boost (radiation directed at peri-tumor area) Dose < 10 or > 20 Gy | It is uncommon for the boost dose to be < 10 or > 20 Gy |
| Total number of lymph nodes with metastases > 5 | It is uncommon for breast cancer to spread to more than 5 lymph nodes. |
